# Nanocomposites Based on PCL and Halloysite Nanotubes Filled with Lysozyme: Effect of Draw Ratio on the Physical Properties and Release Analysis

**DOI:** 10.3390/nano7080213

**Published:** 2017-08-04

**Authors:** Valeria Bugatti, Gianluca Viscusi, Carlo Naddeo, Giuliana Gorrasi

**Affiliations:** Department of Industrial Engineering, University of Salerno, Via Giovanni Paolo II, 132, 84084 Fisciano (Salerno), Italy; vbugatti@unisa.it (V.B.); g.viscusi@studenti.unisa.it (G.V.); cnaddeo@unisa.it (C.N.)

**Keywords:** halloysite nanotubes, PCL, lysozyme, controlled release, active packaging

## Abstract

Halloysite nanotubes (HNTs) were loaded with lsozyme, as antimicrobial molecule, at a HNTs/lysozyme ratio of 1:1. Such a nano-hybrid was incorporated into a poly (*ε*-caprolactone) (PCL) matrix at 10 wt % and films were obtained. The nano-composites were submitted to a cold drawn process at three different draw ratios, *λ* = 3, 4, and 5, where *λ* is *l*_(final length)_/*l*_0(initial length)_. Morphology, physical, and barrier properties of the starting nanocomposite and drawn samples were studied, and correlated to the release of the lysozyme molecule. It was demonstrated that with a simple mechanical treatment it is possible to obtain controlled release systems for specific active packaging requirements.

## 1. Introduction

The expanding consumer demands of minimally-processed fresh, tasty, and convenient food products on an industrial level, is gaining and the food packaging field is experiencing new opportunities for the formulation of novel materials able to with extend the shelf life and control the quality of packaged foods. In the coming years a development of new and alternative food packaging technologies able to control oxidation of foods and microbial contamination is expected, in order to inhibit, limit, or delay microorganisms’ proliferation and the rate of quality decay [1]. The simple blending of low molecular weight antimicrobials in polymer matrices has the disadvantage that the migration and the release of the active molecule cannot be easily predicted and controlled. Foods have peculiar physico-chemical characteristics that could alter the activity of antimicrobial substances; therefore, food components can significantly affect the efficiency of the antimicrobial substances and their release. For instance, the antimicrobial activity and chemical stability of incorporated active molecules could also be influenced by the water activity and the pH of food. The effect of the pH of the release medium has been studied with respect to the lysozyme activity [2] and rate of release from a silica carrier [3]. It was reported that the pH induced conformational changes in lysozyme. The increase of hydrodynamic radius of lysozyme at pH 2 suggested some expansion of the molecules caused by internal repulsive charge interactions. Such an expansion caused the jamming of lysozyme in the pores at acidic pHs [4]. The release of lysozyme from a porous structure can be, then, triggered by the pH, as the lysozyme molecules shrink to their original size at pH 7.

The release kinetics of antimicrobial agents must be able to ensure the concentration above the critical inhibitory dose with respect to the contaminating microorganisms [5]. The phase composition and the morphological organization of the manufacture used for active packaging also play a crucial role with respect to its physical properties and the molecules’ release rate. For example, a possible chain orientation can determine the complex transport phenomena [6,7]. 

Lysozyme is a natural protein and a promising natural antimicrobial agent [8,9,10]. The European Union lists Llysozyme as a food additive (E 1105) with bacteriostatic, bacteriolytic, and bactericidal activity, and the Food and Drug Administration (FDA) considers this molecule as a GRAS (Generally Recognized as Safe). Chemically, it is characterized by a single polypeptide chain, and the antimicrobial activity is related to the capability to hydrolyze the beta 1–4 glycosidic bonds between *N*-acetylglucosamine and *N*-acetylmuramic acid present in peptidoglycans, which constitute 90% of the cell wall of Gram-positive bacteria [11]. Lysozyme has been immobilized on different supports using different methodologies, such as adsorption, entrapment, and surface conjugation [12,13,14], however, its use as a covalently-bonded antimicrobial agent is still very limited. Different strategies have also been used in the literature to modulate the release rate of lysozyme. They are focused on changing the packaging material morphology [15], degree of crosslinking [16,17,18], plasticizer loading [19], nature and amount of additive [20], and number of layers [21]. Some approaches are also focused on the control of the pH of the release medium to modulate its release [22,23]. All these studies show that the release ranges in relatively short times (few days). The goal is to control, as much as possible, the release of lysozyme without changing its chemical structure, and then its activity. 

Very recently, halloysite nanotubes (HNTs) attracted increasing interest as inorganic fillers for polymers. They are cheap green materials and available in thousands of tons from natural deposits. They belong to the alumosilicate clays with a length of about 1000 nm, an internal diameter of 10–15 nm, and external diameters of about 50–80 nm. Their general chemical formula is Al_2_Si_2_O_54_ × *n*H_2_O, and the alumosilicate sheets are rolled into tubes [24,25]. HNTs can be dispersed in polymeric matrices without exfoliation, as required for a good dispersion of layered clays, due to the tubular shape. Polymeric materials have been widely filled with these tubular nano-containers [26,27,28,29] able to release active molecules (antimicrobial, drugs, essential oils, flame retardant, self-healing, anticorrosion, etc.) in particular environments [30,31,32,33,34,35,36,37]. 

In a previous paper we reported the preparation and analysis of novel composites based on poly(lactic acid) (PLA) and HNTs filled with lysozyme for potential in food packaging applications [38]. It is well known that PLA can be applied only in rigid packaging for its brittleness and low elongation at break. In recent years appear very promising biodegradable systems based on blends and copolymers of PLA and PCL to overcome the main drawbacks of both polymers (i.e., brittleness of PLA, and the low modulus and *Tg* of PCL). In this paper we filled PCL with 10% of a HNTs/lysozyme nano-hybrid (HNTs: lysozyme ratio equal to 1:1) and analyzed the physical and release properties. Being that PCL is a biodegradable polyester with a good elongation at break, we also analyzed the possibility to tune the physical and release properties of such active nano-composite through a uniaxially cold draw process. Films were drawn up to *λ* = 3, 4, and 5. The physical properties and the release analysis of lysozyme were studied and correlated to the phase composition and the drawing process. The possibility to apply the same drawing process to copolymers and blends of PLA and PCL, filled with reservoirs of active molecules, like halloysite nanotubes, can help to obtain novel biodegradable active materials with optimal thermal and mechanical properties, and tunable release for specific applications.

## 2. Experimental

### 2.1. Materials

Poly (*ε*-caprolactone) (PCL) Mn 50000 was supplied from Solvay (Solvay Chimica Italia S.p.A, Bollate, Italy). Halloysite nanoclay powders (CAS 1332-58-7), lysozyme powders (CAS 12650-88-3) and tetrahydrofuran (THF) (CAS: 109-99-9) were supplied from Sigma Aldrich (Milano MI, Italy) and used as received.

### 2.2. Preparation of HNTs-Lysozyme 

**Figure 1:**
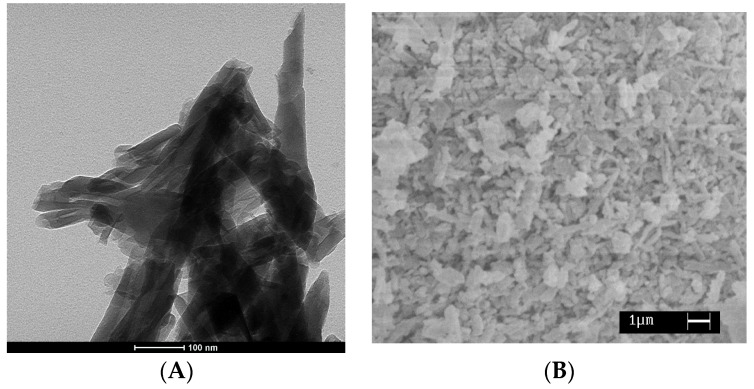
(**A**) TEM image of the pristine HNTs and (**B**) SEM micrograph of the HNTs-lysozyme nanohybrid.

The preparation of the nano-hybrid HNTs-lysozyme was conducted accordingly to a previously reported procedure [38]. Three grams of lysozyme were dissolved in 30 mL of distilled water at 50 °C for 20 min. The HNTs (3 g) were mixed with the lysozyme solution. Then, ultrasonic processing was performed for 10 min, at 40% of amplitude using a UP200S Ultrasonic Processor (Heilscher, Teltow, Germany) to sufficiently disperse the HNTs in the lysozyme solution. The solution was heated at 50 °C for 2 min, then reduced pressure (≅0.085 MPa) was applied to remove the air between and within the hollow tubes. The vacuum was maintained for 15 min. The solution was then taken out from the vacuum and shaken for 5 min. Then vacuum was re-applied to remove the trapped air for 15 min. The HNTs loaded with lysozyme were filtered and dried in an oven for 16 h at 50 °C up to a constant weight. The cycle of vacuum vas then repeated for a second time. The content of lysozyme (wt %) in the HNT-lysozyme hybrid has been calculated according to following Equation, using the TGA analysis:

$$\alpha_{3}=w\cdot \alpha_{1}+\left(1-w\right)\cdot \alpha_{2}$$

where *α*_1_ is the mass loss of lysozyme (99.1%) at 413.2 °C; *α*_2_ is the mass loss of HNTs (16.9%) at 457.9 °C; *α*_3_ is the mass loss of lysozyme/HNTs (58.6%) at 416.0 °C (see TGA Figure 1). Therefore, the content of lysozyme (*w*) in HNTs-lysozyme was estimated to be 50.7%; the HNTs content was 49.3%. 

The evaluated lysozyme amount is too high if compared to the loading capacity of the halloysite nanotubes. This detected content is then relative either to the molecules that were filled into the nanotubes, or to the molecules external to the HNTs, which concurs in different ways to the release (see Section 3).

### 2.3. Nanocomposites Preparation and Draw Processing

Pure PCL and PCL/HNTs-lysozyme, in a weight ratio 90:10, were dissolved in THF at 40 °C for 20 min and sonicated for 10 min at 40% of amplitude. Cast films were hot pressed at 60 °C and cooled to ambient temperature. Strips 1 cm long, were obtained from the films. The strips were put between the clamps of an Instron Dynamometer (Mod 4301, INSTRON Ltd., Norwood, MA, USA), equipped with a temperature chamber. The temperature was fixed to 25 °C and the upper traverse of the dynamometric apparatus was moved at a speed rate of 5 mm/min. Films were drawn at different draw ratios *λ* = *l/l*_0_, where *l* is the final length and *l*_0_ the initial length. The selected values of the draw ratios for all the blends were *λ* = 3, 4, and 5. *λ* = 1 refers to the undeformed nanocomposite.

### 2.4. Methods of Investigation

Bright field transmission electron microscopy (TEM) experiments were performed on a FEI TECNAI G12 Spirit-Twin (120 kV, LaB6) microscope equipped with a FEI Eagle 4k CCD camera (Eindhoven, The Netherlands).

Scanning electron microscopy (SEM) analysis was performed with a LEO 1525 microscope (LEO Electron Microscopy Inc., Thornwood, NY, USA). Thermogravimetric analyses (TGA) were carried out from 30 to 900 °C (heating rate of 10 °C/min) under air flow, using a Mettler TC-10 thermo-balance (Mettler-Toledo GmbH, Greifensee, Switzerland).

Infrared spectra (IR) were recorded in attenuated total reflectance (ATR) mode (Bruker Italia, Milano, Italy) using a Bruker spectrometer, model Vertex 70 (average of 32 scans, at a resolution of 4 cm^−1^).

Mechanical properties were evaluated on all the nanocomposites, using an INSTRON 4301 dynamometric apparatus. The experiments were conducted at room temperature with the deformation rate of 10 mm/min. The initial length of the samples was 10 mm. The mechanical properties, evaluated from stress (*σ*)/strain (*ε*) curves, were: elastic modulus E (MPa), evaluated by applying Hooke’s law (*σ* = E × *ε*) in the interval of deformation less than 0.2%; stress at the yield point *σ_y_* (MPa); elongation at the yield point *ε_y_* (mm/mm %); stress at the break point *σ_b_* (MPa); and elongation at the break point *ε_b_* (mm/mm %) [39]. Data were averaged on five samples.

Barrier properties of water vapor were evaluated using a McBain spring balance system. Samples were suspended from a helical quartz spring supplied by Ruska Industries (Houston, TX, USA) having a spring constant of 1.52 cm/mg. The temperature was controlled to 30 ± 0.1 °C by a constant temperature water bath. Samples were exposed to the water vapor at fixed pressures, *P*, giving different water activities *a* = *P/P*_0_, where *P*_0_ is the saturation water pressure at the experimental temperature. The spring position was recorded as a function of time using a cathetometer. The spring position data were converted to mass uptake data using the spring constant, and the process was followed to a constant value of sorption for at least 24 h. 

Measuring the increase in weight as function of time, for the samples exposed to the vapor at a given partial pressure, it is possible to obtain the equilibrium value of sorbed vapor, *C*_eq, (gsolvent/100 gpolymer)_. In the case of Fickian behavior, when the sorption follows a linear dependence on the square root of time, it is possible to derive the mean diffusion coefficients from the linear part of the reduced sorption curves, reported as *C_t_/C_eq_* versus square root of time, by the Equation (1) [40]:

(1) $$\frac{C_{t}}{C_{eq}}=\frac{4}{d}{\left(\frac{Dt}{\pi }\right)}^{1/2}$$

where *C_t_* is the penetrant concentration at the time *t*, *C_eq_* the equilibrium value, *d* (cm) the thickness of the sample, and *D* (cm^2^/s) is the average diffusion coefficient. 

All the samples showed a Fickian at all the considered activities. Using Equation (1) the diffusion coefficient, *D*, at any vapor activity (*a* = *P/P*_0_) in the range 0–0.6 was derived, as was the equilibrium concentration of solvent into the samples, *C_eq_*_, (gsolvent/100 gpolymer)_. For polymer-solvent systems, the diffusion parameter is usually not constant, but depends on the vapor concentration, according to the empirical Equation (2):

*D* = *D*_0_*exp*(*γC_eq_*) (2)

where *D*_0_ (cm^2^/s) is the zero concentration diffusion coefficient (related to the fractional free volume and to the morphology of the material), and *γ* is a coefficient which depends on the fractional free volume and on the effectiveness of the penetrant to plasticize the matrix. 

The release kinetics of the lysozyme were followed using a UV-2401 PC Spectrometer (Shimadzu, Kyoto, Japan). The tests were performed using rectangular specimens of 4 cm^2^ and the same thickness (150 μm), placed into 25 mL physiological solution and stirred at 100 rpm in an orbital shaker (VDRL MOD. 711+, Asal S.r.l., Milan, Italy). The release medium was withdrawn at fixed time intervals and replenished with fresh medium. The considered band was at 265 nm. 

## 3. Results and Discussion

Figure 1A reports the TEM image of the pristine HNTs. It is evident a tubular structure where the average length of the tubes ranges between 0.5–1 μm, the inner diameter ranges between 10–15 nm, and outer diameters between 100–200 nm. Figure 1B shows SEM micrograph of the prepared halloysite nano-hybrid. In the used experimental conditions, it is evident a significant amount of lysozyme crystals outside the tubes. 

**Figure 2:**
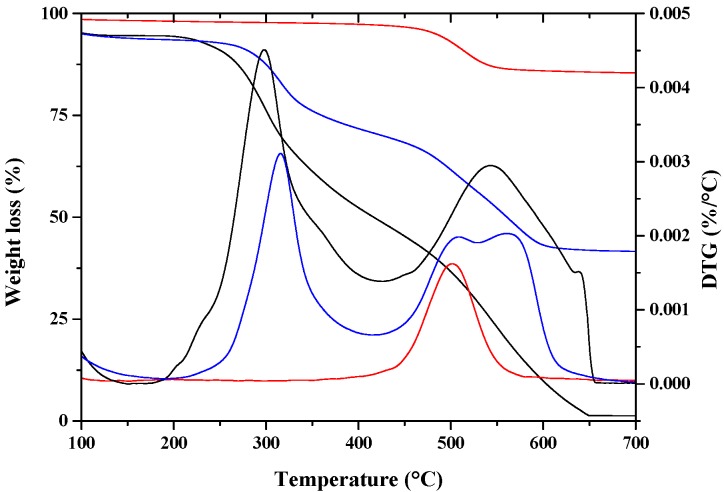
TGA/DTG of: HNTs (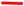), Lysozyme (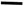), nano-hybrid HNTs/Lysozyme (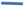).

The distribution of active molecules inside and/or around HNTs nano-hybrids is very complex to be accurately determined. The degradation temperatures of the single components and the nano-hybrid has been demonstrated to be a useful tool to have an indication about the molecules’ intercalation efficiency [41]. Figure 2 reports the mass loss (%) (TG) and derivative thermogravimetry (DTG) evaluated on HNTs, lysozyme and HNTs-lysozyme. Halloysite shows one degradation step at about 500 °C, which is attributed to the dehydroxylation of the matrix [42]. The thermal degradation of lysozyme occurs in two main steps. The first one, centered at about 298 °C, is related to the breaking of the amide bonds. The second one, centered at about 542 °C is due to the formation and elimination of low molecular weight volatile oxygenated compounds. A shift toward higher values for the two degradation temperatures of lysozyme, centered at 316 and 566 °C is clearly evident in the nano-hybrid. The degradation of the HNTs does not change going from the crude material to the nano-hybrid. As evidenced from SEM analysis, a part of lysozyme molecules is external to the tubes and degrades at the degradation temperature of the free lysozyme. The lysozyme molecules that show a delay in the degradation were successfully entrapped into the nanotubes and their degradation occurs only after a spillage from the nanotubes. 

**Figure 3:**
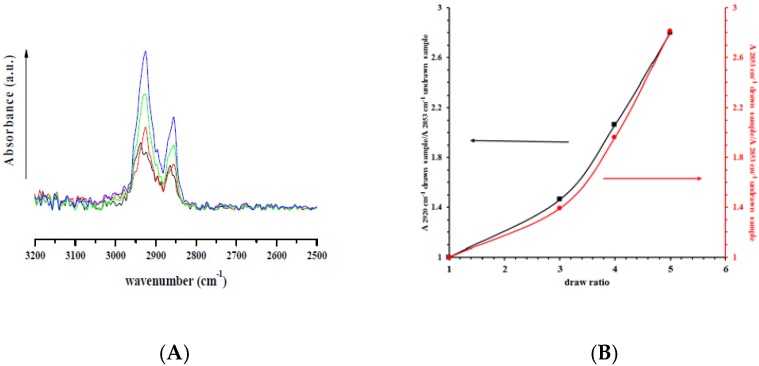
ATR spectra of (**A**): Undeformed nanocomposite (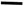), drawn at *λ* = 3 (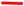), drawn at *λ* = 4 (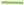), and drawn at *λ* = 5 (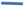); and (**B**) the ratio between absorbance bands of symmetrical (2850 cm^−1^) and asymmetrical (2930 cm^−1^) C–H stretching for drawn samples divided by the absorbance of undrawn samples.

Figure 3A reports the ATR spectra for all nanocomposites, undeformed and drawn at different draw ratios, in the range 2500–3200 cm^−1^. In these regions no absorption bands are visible neither for HNTs nor for lysozyme. The bands at 2850 and 2930 cm^−1^ are related to the symmetrical and asymmetrical stretching from the bonds C–H of the PCL aliphatic chains [43]. It is evident that both bands increase with the draw ratio. Figure 3B reports the intensities of these bands in the drawn samples, divided by the intensity of the undeformed sample. It is evident a correlation between draw ratio and the symmetrical and asymmetrical stretching of the C–H bonds of the aliphatic PCL chains.

**Figure 4:**
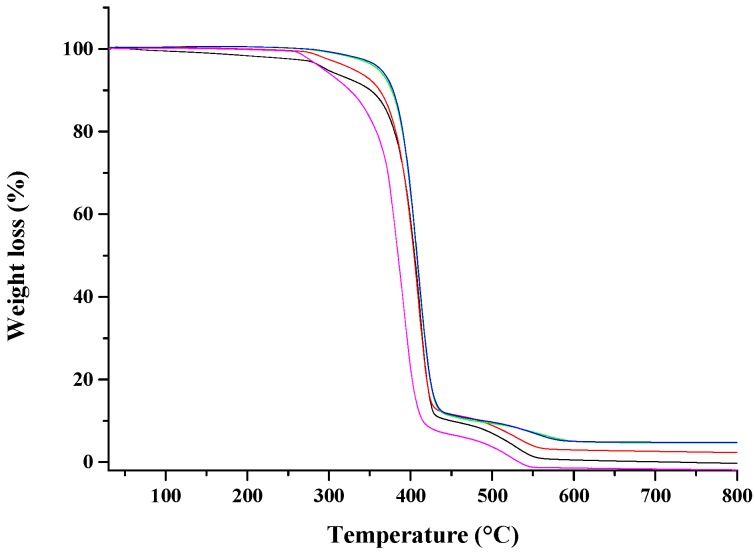
TGA of: PCL (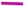), undeformed nanocomposite (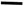), drawn at *λ* = 3 (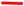), drawn at *λ* = 4 (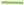), and drawn at *λ* = 5 (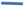).

Figure 4 shows the TGA analysis on the considered nanocomposites, either undrawn or drawn at different draw ratios. For comparison it is reported also the TGA of unfilled PCL and PCL drawn at *λ* = 5. The thermogram of neat PCL exhibits two peaks at about 385 and 480 °C, which evidenced a two-step mechanism for the PCL degradation [44]: (1) random chain scission induced by pyrolysis of the ester groups, with the release of hexanoic acid, CO_2_ and H_2_O, and (2) cyclic monomer of *ε*-caprolactone formation, as a result of unzipping depolymerization process. The drawing process does not significantly affect the thermal degradation of PCL (sample PCL drawn at *λ* = 5 in Table 1), while the incorporation of the nano-hybrid at 10 wt % of HNTs-lysozyme generates an improvement on thermal stability of PCL, which is reflected also in the drawn samples. Degradation temperatures, evaluated at 10%, 50%, and 95% of weight loss for all samples, are reported in Table 1. It has been demonstrated that HNTs in HNTs/polymer nanocomposites are responsible for an improvement in thermal stability [29,45]. As already found, it is hypothesized that the HNTs dispersed into the matrix, act as a barrier to the mass transport slowing down the escape of the volatile products during the degradation process [46]. 

Mechanical properties were conducted on all nanocomposites, either undeformed or monoaxially drawn samples. Table 2 reports the mechanical parameters for all samples, evaluated from the stress-strain curves here not reported. For comparison, the mechanical parameters for unfilled PCL are also reported, taken from a previous work [47]. It is also reported the mechanical behavior of unfilled PCL drawn at *λ* = 5. It is evident that either the introduction of the nano-hybrid, or the drawing process, determine a reinforcement of the PCL matrix. In the case of composite systems, the enhancement of the mechanical properties of nanocomposites requires a high degree of load transfer between continuous and the dispersed phases. If the interfacial adhesion between the phases is weak, the filler behaves as holes or nanostructured flaws, introducing local stress concentrations, and its benefits on the properties are lost [48]. The filler must be well dispersed because, in the case of poor dispersion, the strength is significantly reduced. Furthermore, solid state drawing of nanocomposites is largely dominated by regions of a lower HNTs volume fraction and this could certainly alter both orientation and distribution of HNTs along the length of the fiber. The improvement of the mechanical parameters is evident for the composite fibers, in particular at higher draw ratios. This could be attributed to highly-dispersed and well-aligned HNTs. The only parameter that results in a decrease, either with filler loading or with the draw ratio, is the elongation at break. This is quite expected because of the incompatibility between the two phases due to the different chemical nature of both, more evidenced at higher draw ratios where a stronger phase separation can occur for the mechanical treatment.

**Figure 5:**
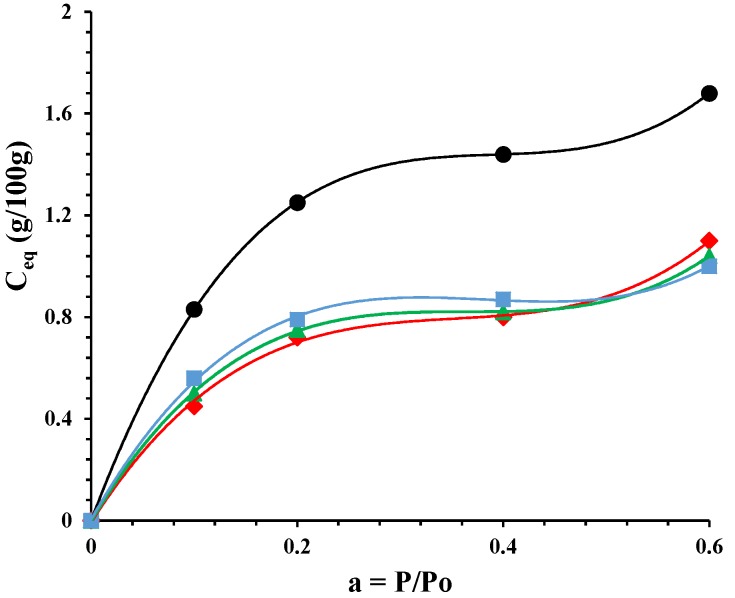
Sorption isotherms for: PCL/10%HNTs-lysozyme (•), a sample drawn at *λ* = 3 (♦), a sample drawn at *λ* = 4 (▲), and a sample drawn at *λ* = 5 (■).

Figure 5 reports the sorption isotherms of the undeformed nanocomposite and the ones drawn at different draw ratios. The mode of sorption, for all the samples, can be interpreted with a dual-mode mechanism. Sorption is visualized as a process in which there are dual modes: at low activity the penetrant molecules are normally dissolved and free to diffuse, or they are immobilized on polar sites of the polymer matrix. At higher activities the increase of sorption is due to the water plasticization effect on the material. It is evident that a decrement of sorption for all drawn samples in the whole investigated activity range. The lowering of water sorption is quite independent of the draw ratio. The morphological organization of the drawn samples, in terms of the amount of the permeable phase, is similar in the fibers. The drawing process either induces further crystallization in the PCL or allows an alignment in the polymeric chains that can represent impermeable domains to water sorption. 

The analysis of sorption and diffusion of low molecular weight molecules are of great importance in determining the applications of nanocomposites materials in the packaging field. Modulating the nanocomposite’s composition, in terms of the nature of single components, loading, and morphology, it is possible to project novel and active materials for the required uses. In order to determine how the composition of the multiphase materials, drawn at different draw ratios, affects the transport properties of the PCL matrix, sorption (thermodynamic parameter) and diffusion (kinetic parameter) for all the samples using water vapor were evaluated.

**Figure 6:**
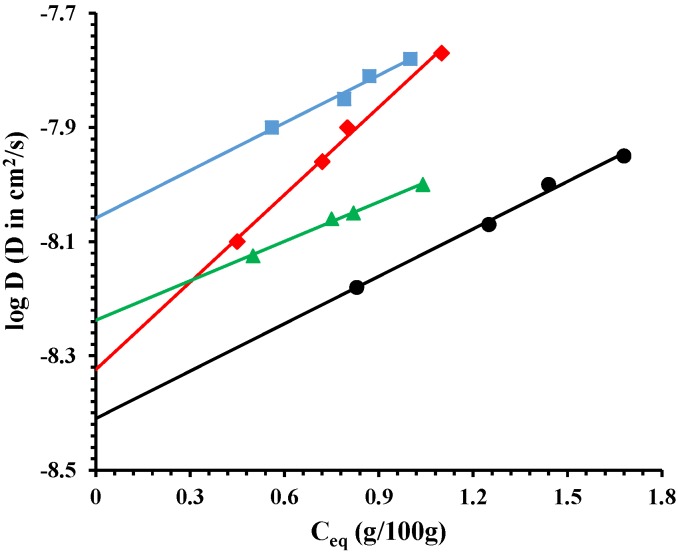
Log diffusion for: PCL/10%HNTs-lysozyme (●), a sample drawn at *λ* = 3 (♦), a sample drawn at *λ* = 4 (▲), and a sample drawn at *λ* = 5 (■).

Figure 6 reports the log of diffusion, *D* (cm^2^/s), as a function of *C_eq_* (g/100 g) of sorbed water vapor. It is evident a slight increase of the diffusion coefficient with the draw ratio. This can be due to micro-voids formed during the drawing process. It is worth noting, as for the mechanical properties, that the two phases (organic-inorganic) are not perfectly miscible. The drawing process, at ambient temperature, can generate a slight disconnection between the two phases, evidenced by the diffusion. The small molecules can diffuse through mechanically-induced microvoids.

**Figure 7:**
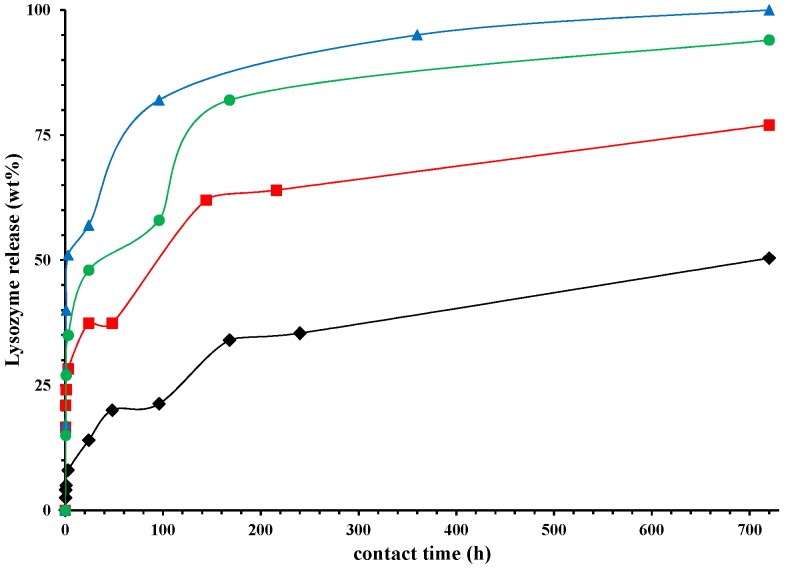
Lysozyme released (wt %), as function of time (hours) for: PCL/10%HNTs-lysozyme (⧫), a sample drawn at *λ* = 3 (■), a sample drawn at *λ* = 4 (●), and a sample drawn at *λ* = 5 (▲).

**Figure 8:**
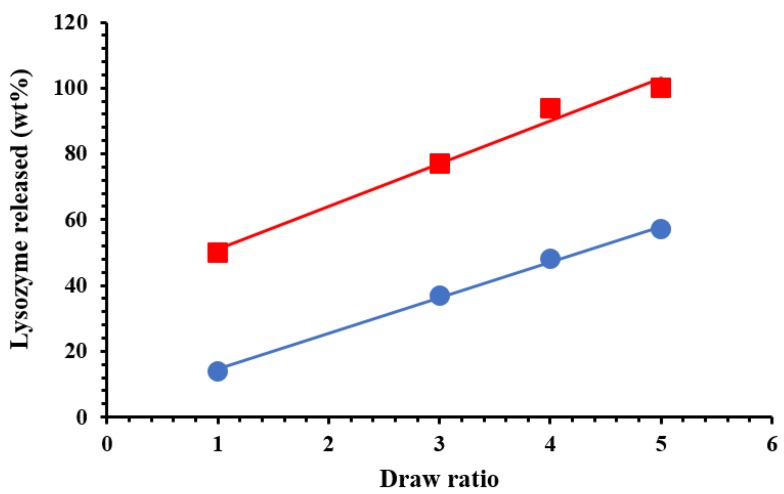
Lysozyme released (wt %) after: 24 h (●) and one month (■).

Figure 7 reports the release of lysozyme (in wt %) in physiological solution, as a function of time (hours). In order to demonstrate the capability of HNTs to slow down the lysozyme release, we prepared also a sample in which the same amount of lysozyme (5 wt %) is free dispersed in PCL film. We observed that in the first 24 h about the 90% of lysozyme is released, while it is evident from Figure 7 that only the 25% is released from the nano-composite in which the molecule is entrapped into the HNTs. After 720 h the lysozyme release from the nano-composite is around 50%. The release of Lysozyme from the nano-composites is a complex phenomenon. It is evident a first stage of fast release in the first 24 h could be due to the free lysozyme molecules located on the surface of the sample of free to diffuse through the microvoids mechanically inducted (see discussion on the diffusion). The second stage, from 24 to about 60 h, could be related to the diffusion of the molecules from the bulk and/or the HNTs, the third stage represents the spillage of the most entrapped lysozyme molecules. The higher is the draw ratio, the higher the lysozyme (wt %) capable to exit from the nanocomposites. After one month (720 h) the lysozyme is released for 100% in the case of a sample drawn at *λ* = 5, for 94% and 77% for the nanocomposites drawn at *λ* = 4 and 3, respectively, and only for 50% for the undeformed sample. Figure 8 reports the Lysozyme release (wt %) at two different times: 24 h and one month. It is evident a linear increase of the active molecule released, as function of the draw ratio, for both the chosen times. In the case of food packaging application 1 month is a long time of storage for fresh foods, and in this time it is desirable that 100% of the antimicrobial molecule is released. In particular, in the case of meat, it has been found that a lower burst and a slower release is preferable, like for the sample drawn at *λ* = 3 [49], while for foods rich of water, like tomatoes, strawberries, or grapefruit [50], an initial fast release around 50%, followed by a slower step of release is better, like for the samples drawn at *λ* = 4 and 5.

## 4. Concluding Remarks

In this work we investigated the influence of the mechanical drawing on nanocomposites composed of poly (*ε*-caprolactone) (PCL) filled with 10% of HNTs/Lysozyme nano-hybrid. Films were uniaxially cold drawn up to *λ* = 3, 4, and 5. The physical properties and the release analysis of lysozyme were studied and correlated to the phase composition and the drawn ratio.

From ATR measurements it was demonstrated that the bands at 2850 and 2930 cm^−1^, related to the symmetrical and asymmetrical stretching from the bonds C–H of the PCL aliphatic chains, increased with the draw ratio. 

The incorporation of the nano-hybrid at 10 wt % of HNTs-lysozyme allowed an improvement of thermal stability of PCL, which was also reflected in the drawn samples.

Mechanical properties demonstrated that the strength of the samples were improved, in particular, at higher draw ratios. This was attributed to highly-dispersed and well-aligned HNTs. The elongation at break resultedly decreased because of the incompatibility between the two phases due to the different chemical nature of both, more evidenced at higher draw ratios where a stronger phase separation occurs for the mechanical treatment.

Barrier properties to water vapor showed that sorption decreased for all drawn samples, while the diffusion slightly increased with the draw ratio. This was attributed to possible micro-voids formed during the drawing process.

The release kinetics of lysozyme in physiological solution showed, in accordance with diffusion data, an increasing of the release with increasing the draw ratio. It was demonstrated that with a mechanical treatment it is possible to obtain nanocomposites at controlled release for specific active packaging requirements.

Work is in progress in order to test the efficiency of such systems with respect to *Bacillus sp.* considering the effect of the solvent treatment, the temperature used for hot pressing the material, and the drawing process. 

## Figures and Tables

**Table 1:** Thermal data for all nanocomposites and unfilled PCL, evaluated from TGA.

Sample	*T_d_* (10% Weight Loss)	*T_d_* (50% Weight Loss)	*T_d_* (95% Weight Loss)
PCL	320 °C	385 °C	480 °C
PCL drawn at *λ* = 5	325 °C	390 °C	505 °C
PCL/10%HNTs-Lysozyme	350 °C	403 °C	518 °C
Nano-composite drawn at *λ* = 3	360 °C	405 °C	537 °C
Nano-composite drawn at *λ* = 4	373 °C	407 °C	573 °C
Nano-composite drawn at *λ* = 5	377 °C	409 °C	583 °C

**Table 2:** Mechanical properties for all nanocomposites and unfilled PCL, evaluated from stress-strain curves.

Sample	*E* (MPa)	*σ_y_* (MPa)	*ε_y_* (%)	*σ_b_* (MPa)	*ε_b_* (%)
PCL ^#^	185 ± 24	9.95 ± 0.23	11.50 ± 3.1	15.88 ± 0.23	616 ± 14.22
PCL drawn at *λ* = 5	530 ± 22	36.45 ± 0.67	13.57 ± 3.4	32.52 ± 0.34	320 ± 15.34
PCL/10%HNTs-Lysozyme	320 ± 16	10.24 ± 0.34	8.79 ± 3.7	18.14 ± 0.65	570 ± 12.31
Nano-composite drawn at *λ* = 3	335 ± 15	16.72 ± 0.46	9.31 ± 2.4	28.98 ± 0.47	157 ± 16.26
Nano-composite drawn at *λ* = 4	347 ± 23	23.60 ± 0.42	14.08 ± 3.6	36.10 ± 0.36	162 ± 8.420
Nano-composite drawn at *λ* = 5	447 ± 20	39.40 ± 0.57	14.06 ± 4.8	54.61 ± 0.74	102 ± 13.27

^#^ Data from ref. [47].
